# Effect of Online Mindfulness‐Based Stress Reduction on Psychological Distress and Pregnancy Rate in Infertile Women Undergoing In Vitro Fertilization

**DOI:** 10.1155/ogi/1249143

**Published:** 2026-05-04

**Authors:** Ramin Shiraly, Afra Khani Valizadeh, Sedigheh Amooee, Mozhgan Moghtaderi, Seyed Hesamedin Nabavizadeh, Farshad Nazaraghaie, Sara Mostafavi

**Affiliations:** ^1^ Department of Preventive and Community Medicine, School of Medicine, Shiraz University of Medical Sciences, Shiraz, Iran, sums.ac.ir; ^2^ Student Research Committee, Shiraz University of Medical Sciences, Shiraz, Iran, sums.ac.ir; ^3^ Department of Obstetrics and Gynecology, Infertility Research Center, Shiraz University of Medical Sciences, Shiraz, Iran, sums.ac.ir; ^4^ Allergy Research Center, Shiraz University of Medical Sciences, Shiraz, Iran, sums.ac.ir; ^5^ Social and Individual Stress Reduction Association, Shiraz, Iran

**Keywords:** infertility, in vitro fertilization, meditation, mindfulness, psychological distress

## Abstract

**Objectives:**

This study evaluated whether an online mindfulness‐based stress reduction (MBSR) program could reduce psychological distress and improve pregnancy rates in women undergoing in vitro fertilization (IVF).

**Methodology:**

A total of 133 infertile women were randomized to an online MBSR intervention or a control group. The intervention included twice‐weekly mindfulness sessions over 3 months. Primary outcomes were anxiety and depression, and the ultrasound‐confirmed intrauterine pregnancy rate was a secondary outcome.

**Results:**

The intervention group showed significant reductions in anxiety and a significant borderline decrease in depression compared to controls. Participation in MBSR was also associated with a higher rate of ultrasound‐confirmed intrauterine pregnancy. Online MBSR may effectively reduce psychological distress in women undergoing IVF, with potential benefits for pregnancy outcomes.

**Conclusion:**

This study suggests that online MBSR may serve as an effective complementary approach for alleviating emotional distress and potentially enhancing pregnancy outcomes in women undergoing IVF. Online MBSR programs appear to be a valuable tool for supporting women in managing infertility‐related psychological challenges.

**Trial Registration:** Iranian Registry of Clinical Trials (IRCT): IRCT20241002063238N1

## 1. Introduction

Infertility‐related psychological distress is common among couples undergoing evaluation and treatment for infertility. Emotional reactions such as anxiety, sadness, frustration, and a sense of loss frequently arise after an infertility diagnosis and may progress to depression, reduced self‐esteem, stigma, and social isolation [1, 2]. Women often experience heightened distress during assisted reproductive treatments due to fear of failure, repeated procedural burdens, and the substantial financial costs associated with assisted reproductive technology (ART) [1].

Clinically, infertility is defined as the failure to conceive after 12 months of regular unprotected intercourse and may be classified as primary or secondary. Globally, an estimated 48 million couples are affected, with a disproportionately high burden in developing nations where access to reproductive and mental health services remains limited [3, 4]. Mental health problems are reported in 25%–60% of infertile women, particularly in low‐ and middle‐income settings [5]. Accordingly, both the World Health Organization (WHO) and the National Institutes of Health (NIH) emphasize the need for effective strategies to prevent and manage infertility‐related psychological distress [1].

Psychological stress may negatively influence reproductive outcomes during ART, suggesting that supportive interventions could play a dual role in improving emotional well‐being and potentially enhancing pregnancy rates [6, 7]. Behavioral and mind–body approaches, including cognitive behavioral therapy (CBT), yoga, and mindfulness‐based interventions, have shown promise in reducing distress among infertile populations [8, 9]. Mindfulness‐based stress reduction (MBSR) is a structured program that cultivates nonjudgmental awareness of present‐moment experiences to improve emotional regulation and overall mental well‐being [10, 11]. Meditation practices within MBSR aim to enhance attention, acceptance, and cognitive flexibility and have been increasingly applied for stress management and psychological resilience across diverse clinical groups [12–14].

Although there is growing interest in mind–body interventions for women undergoing ART, the evidence on their effectiveness in reducing psychological distress and improving pregnancy outcomes remains limited and inconsistent. Some recent systematic reviews and meta‐analyses have reported positive effects on anxiety, depression, and pregnancy rates, but many studies are small, heterogeneous, and vary widely in intervention type and quality, resulting in mixed findings and methodological limitations [15–18]. Therefore, this study aimed to evaluate the impact of an online MBSR program on anxiety, depression, and pregnancy rates among Iranian women undergoing in vitro fertilization (IVF).

## 2. Materials and Methods

### 2.1. Study Design

This parallel group randomized controlled trial was conducted among infertile women referred for ART at Zeinab Hospital Infertility Clinic, the largest infertility center in Southern Iran, during the second half of 2022. Infertility was defined as failure to achieve pregnancy after at least 12 months of regular unprotected intercourse. During the study period, 223 women attended the clinic and were screened for eligibility criteria. No patients or members of the public were involved in the design, conduct, reporting, or dissemination plans of this study. No changes were made to the trial protocol after study commencement. All participants provided written informed consent in accordance with institutional guidelines and the Declaration of Helsinki.

### 2.2. Inclusion and Exclusion Criteria

Women were eligible for participation if they were aged 20–45 years, had a physician‐confirmed diagnosis of infertility, had a body mass index (BMI) between 19.8 and 29 kg/m^2^, were able and willing to participate in online mindfulness meditation sessions, and could understand and complete the study questionnaires. Participants were excluded if they were currently using fertility‐related medications (such as hormonal treatments or herbal remedies) within the past 3 months or had undergone surgical infertility treatments within the past year. Women who had engaged in alternative or complementary therapies, including yoga, meditation, or acupuncture, within the 12 months preceding the study were also excluded. Individuals with a history of serious psychiatric disorders, including psychotic disorders, bipolar disorder, or major depressive disorder, or those currently taking antidepressant or antianxiety medications, were not eligible. Additional exclusion criteria included the presence of debilitating chronic diseases, a history of tobacco smoking or substance abuse, and an infertility duration exceeding 15 years.

### 2.3. Sampling and Randomization

Eligible women referred to Zeinab Hospital Infertility Clinic for assisted reproductive treatment between June and December 2022 were consecutively screened for participation in the study. The minimal sample size was determined using the formula for comparing two means. Assuming a confidence interval of 95%, a power of 80% to detect differences, and a moderate effect size of 0.55, the minimum required sample size was calculated as 106 patients (53 in each group). A total of 133 eligible participants were randomly assigned in a 1:1 ratio to either the MBSR intervention group (*n* = 67) or the control group (*n* = 66) using a computer‐generated randomization sequence. The sequence was created by a researcher not involved in participant recruitment or outcome assessment to ensure allocation concealment. Assignments were placed in sequentially numbered, opaque, sealed envelopes, which were opened only after participants completed baseline assessments. Participant enrollment and assignment were conducted by study staff who were unaware of the allocation sequence. The MBSR group received online MBSR sessions in addition to routine infertility care, which included standard IVF protocols, hormonal monitoring, and physician counseling according to clinic guidelines. The control group received routine infertility care alone without any additional psychological intervention. Due to the nature of the intervention, participant blinding was not possible, and the trial was conducted as an open‐label study; however, outcome assessors performing pregnancy ultrasounds were blinded to group allocation.

### 2.4. Intervention

Participants in the intervention group received an online MBSR program in addition to routine infertility care. The program consisted of 16 sessions conducted over eight weeks, with two sessions per week. Each session lasted 1.5–2 h and was delivered via Google Meet by a certified meditation instructor. Participants were encouraged to practice mindfulness techniques at home for at least 20 min daily, including mindful breathing, body scan exercises, or a combination of both. All sessions were supervised by one of the study authors (F.N.), a MBSR expert and experienced member of the Shiraz Meditation and Yoga Academy, to ensure consistency and adherence to the intervention protocol. Attendance was recorded for each session, and participants were followed regularly to encourage adherence to daily practice. The control group received routine infertility care alone, which included standard IVF protocols, hormonal monitoring, and physician counseling, without any additional psychological intervention.

### 2.5. Outcomes

The primary outcomes of the study were psychological distress measures, including depression and anxiety. Depression was assessed using the validated Persian version of the Beck Depression Inventory‐II (BDI‐II), and anxiety was assessed using the Persian State–Trait Anxiety Inventory (STAI). Both questionnaires were self‐administered online via Google Forms at two time points: baseline (before the start of the intervention) and at the end of the 8‐week intervention period. Depressive symptoms were assessed using BDI‐II. BDI‐II is a widely used self‐report tool for identifying and assessing the severity of depressive symptoms [19]. The scale consists of 21 items that measure common symptoms of depression over the past two weeks. The severity of symptoms is rated on a Likert scale ranging from 0 to 3 [20]. The validity and reliability of the Persian version of the BDI‐II scale have been confirmed [21]. Anxiety was measured using the STAI scale. The scale assesses both state (current) anxiety and trait (stable over time) anxiety. Each subscale consists of 20 items, and each item is rated on a 4‐point Likert scale [22]. The Persian version of the scale was validated, and the Cronbach’s alpha internal consistency was calculated to be 0.94 [23]. The secondary outcome was that pregnancy rate, defined as ultrasound‐confirmed intrauterine pregnancy at 6‐7 weeks after embryo transfer. Ultrasound examinations were performed by clinicians blinded to group allocation to minimize bias. The timing of the intervention was coordinated with the participants’ IVF cycle, ensuring that the 8‐week MBSR program overlapped with the period from ovarian stimulation through embryo transfer.

### 2.6. Data Analysis

Data were analyzed using SPSS Version 26. An exploratory analysis was conducted for two main reasons. First, given the inevitability of noncompliance and its potential to yield incomplete or unreliable data on the primary outcomes (BDI and STAI scores), participants who did not complete the postintervention questionnaires were excluded from the outcome analysis. Second, the primary analysis indicated that participants who dropped out did not differ systematically from those included with respect to baseline study characteristics. Accordingly, the analyses followed a modified intention‐to‐treat (mITT) approach, including all randomized participants with available outcome data (complete‐case analysis). Continuous variables were assessed for normality using the Shapiro–Wilk test. Within‐group differences between baseline and postintervention scores were evaluated using the Wilcoxon signed‐rank test, while between‐group comparisons were performed using the Mann–Whitney *U* test. The state and trait subscales of the STAI were analyzed separately. Categorical variables, including clinical pregnancy rate, were compared using Pearson’s chi‐square test. All tests were two‐sided, and a *p* value < 0.05 was considered statistically significant.

## 3. Results

**FIGURE 1:**
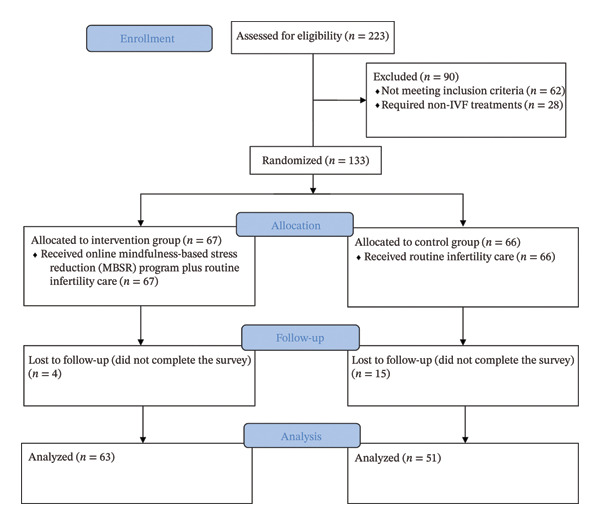
Consolidated Standards of Reporting Trials (CONSORT) flowchart.

Of the 223 women assessed for eligibility, 133 (59.6%) were enrolled, while 90 (40.4%) were excluded during the screening process. Among the screen failures, 45 did not meet eligibility criteria and 17 declined participation. In addition, 28 women were redirected to fertility treatments other than IVF, such as surgical procedures or intrauterine insemination (IUI), following clinical evaluation. The flow of participants throughout the study is summarized in Figure 1.

A total of 133 eligible women were randomized; however, 19 participants discontinued the study for various reasons during the follow‐up period, resulting in a final analytic sample of 114 subjects. The mean age of participants was 35.2 ± 5.4 years, and the mean duration of infertility was 7.9 ± 4.0 years. More than one‐third (37%) held a college or university degree, and over 80% presented with either a female‐factor or mixed‐pattern infertility diagnosis. Baseline demographic and clinical characteristics of the intervention and control groups are presented in Table 1. No statistically significant differences were observed between groups at baseline.

**TABLE 1:** Baseline demographic, psychological, and infertility‐related characteristics of the participants.

Variable	MBSR^∗^ intervention group (*n* = 63)	Control group (*n* = 51)	*p* value
Age (y, mean ± SD)	35.63 ± 5.40	34.76 ± 5.46	0.564
Education, no. (%)			0.368
Primary school	10 (15.9)	13 (25.5)	
High school diploma	30 (47.6)	19 (37.3)	
University degree	23 (36.5)	19 (37.3)	
Infertility duration (y, mean ± SD)	7.85 ± 3.80	8.13 ± 4.29	0.866
Causes of infertility, no. (%)			0.128
Male factor	6 (9.5)	13 (25.5)	
Female factor	34 (54.0)	20 (39.2)	
Mixed	21 (33.3)	16 (31.4)	
Unknown	2 (3.2)	2 (3.9)	
Depression Score (mean ± SD)	12.76 ± 11.10	12.39 ± 5.25	0.230
State Anxiety Score (mean ± SD)	44.84 ± 10.95	43.80 ± 9.98	0.791
Trait Anxiety Score (mean ± SD)	42.80 ± 10.09	42.74 ± 9.87	0.968

^∗^MBSR: mindfulness‐based stress reduction.

### 3.1. Primary Outcomes: Psychological Distress

Comparisons of psychological distress outcomes across groups are shown in Table 2. After the 3‐month follow‐up, participants in the MBSR group demonstrated significantly greater reductions in state anxiety (*p* < 0.001) and trait anxiety (*p* = 0.020) compared with controls. Whereas mean BDI‐II scores increased over time in the control group, the MBSR group exhibited a borderline significant reduction in depressive symptoms (*p* = 0.057). Similar to what was observed in the within‐group analysis, between‐group comparisons after the 3‐month follow‐up demonstrated significant differences between the MBSR and control groups across all psychological distress measures. Specifically, participants in the MBSR group had a lower mean state anxiety score (41.26 ± 10.49) compared to the control group (45.72 ± 9.69), which was statistically significant (*Z* = −2.21, *p* = 0.029). Similarly, trait anxiety scores were significantly lower in the MBSR group (40.22 ± 10.07) than in the control group (44.70 ± 8.34; *Z* = −2.19, *p* = 0.012). Furthermore, BDI‐II scores were also significantly reduced in the MBSR group (11.51 ± 8.39) compared with the control group (15.01 ± 7.14; *Z* = −2.99, *p* = 0.003).

**TABLE 2:** Within‐group changes in psychological distress before and after intervention among the participants.

MBSR group	Measure	Mean ± SD	*t*	*p* value
Control group	State Anxiety Score			
	Before	44.84 ± 10.95	−3.36	*p* < 0.001
	After	40.71 ± 8.35		
	Trait Anxiety Score			
	Before	42.80 ± 10.09	−2.32	*p* = 0.020
	After	40.22 ± 10.07		
	Depression Score			
	Before	12.76 ± 11.10	−1.90	*p* = 0.057
	After	11.50 ± 8.39		
	State Anxiety Score			
	Before	43.80 ± 9.98	−0.91	*p* = 0.364
	After	44.35 ± 9.18		
	Trait Anxiety Score			
	Before	42.74 ± 9.87	−2.11	*p* = 0.034
	After	44.70 ± 8.34		
	Depression Score			
	Before	12.39 ± 5.25	−3.360	*p* = 0.001
	After	15.01 ± 7.14		

Abbreviation: MBSR, mindfulness‐based stress reduction.

### 3.2. Secondary Outcome: Ultrasound‐Confirmed Intrauterine Pregnancy

**FIGURE 2:**
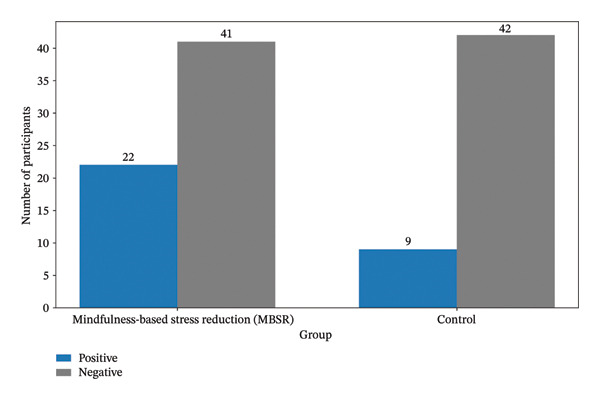
Comparison of ultrasound‐confirmed intrauterine pregnancy between mindfulness‐based stress reduction (MBSR) and the control group.

Regarding the secondary outcome, a significantly higher proportion of women in the MBSR group had ultrasound‐confirmed intrauterine pregnancy compared with the control group (*p* = 0.031). Furthermore, the odds of obtaining an ultrasound‐confirmed intrauterine pregnancy were 2.5 times higher among participants who completed MBSR sessions compared with those who did not (OR = 2.5; 95% CI: 1.03–6.07), as shown in Figure 2.

### 3.3. Adverse Events

No adverse events related to the online MBSR intervention were reported during the study. Participants tolerated the intervention well, and no participants discontinued the study due to intervention‐related issues.

## 4. Discussion

The present study examined the efficacy of an online MBSR education on emotional distress and pregnancy rate of women undergoing IVF. The study found that using mindfulness meditation‐based intervention with ongoing home practices would ameliorate psychological distress and would be associated with a higher pregnancy rate in infertile women who completed an IVF treatment program. This finding is in line with previous research indicating positive consequences of the use of mindfulness‐based interventions on emotional state and pregnancy rate of infertile women [24]. Regarding depressive syndromes, the present study found an increase in BDI‐II scores in the control group and a borderline decrease in the MBSR group over the follow‐up period. A previous interventional study by Kalhori et al., which examined the effect of mindfulness‐based group counseling on the mood state of infertile women during IVF, found that while depressive symptoms scores increased in the control group, they decreased significantly in the intervention group [25]. This finding may indicate that infertile women who experience a treatment failure may develop depressed mood regardless of prior psychiatric history, and mindfulness‐based interventions may prevent or relieve such emotional responses and mood changes. The present study findings also revealed a more explicit effect of MBSR intervention on anxiety than depression in the target group. This may be because MBSR enhances one’s awareness of the present moment, leading to more successful emotion regulation, and helps reduce perceived stress and anxiety [12]. Moreover, results of a recent systematic review by Kundarti et al. demonstrated that while mindfulness‐based cognitive therapy (MBCT) targets depressive symptoms, MBSR specifically targets stress and anxiety among infertile women [9]. It has been shown that other closely related mindfulness practices, such as Mindful Self‐Compassion and Yoga, may help enhance positive emotional states and reduce psychological distress in women undergoing ART [26, 27].

The present study used the STAI scale to evaluate participants’ anxiety. Our findings indicated that MBSR intervention had a positive effect on both state and trait anxiety. It should be noted that the STAI scale score output may reflect the emotional state of the participants and do not necessarily indicate the presence of a mental health disorder, as confirmation of the latter needs assessment of mental state by a psychiatrist or a licensed mental health professional. Nevertheless, the state and trait anxiety can reflect transient anxiety states in healthy individuals, where state anxiety represents a more temporary and situational reaction to anxiety‐provoking events, and trait anxiety represents a more sustained feeling of fear and premorbid anxiety over time [28].

Current knowledge on the relationship between mindfulness‐based interventions and pregnancy rates among women undergoing infertility treatment is more contradictory and less established. Some previous studies have reported little or no significant effect of psychosocial and behavioral interventions on the pregnancy rate of women undergoing ART [15, 29]. The present study findings suggest a positive effect of MBSR on the pregnancy rate in the target group. Similar to our results, Li et al. indicated that women who received mindfulness‐based intervention during infertility treatments had greater pregnancy rates compared to the control group [24]. A meta‐analysis evaluating the effects of psychological interventions on infertile women reported that such interventions did not significantly improve mental health outcomes, although there was some evidence suggesting a beneficial effect on pregnancy rates [30]. Assuming a positive effect of mindfulness‐based and other related stress‐reductive interventions on pregnancy rates in women undergoing ART, this benefit has been plausibly explained in biological pathway terms. The hypothalamic–pituitary–adrenal axis (HPA) is a critical component in the context of infertility, as it regulates hormones such as cortisol, which can adversely affect pregnancy rates, particularly in ART such as IVF treatment [31]. Hypersecretion of cortisol related to anxiety has been reported as prevalent among infertile women [32]. A recent systematic review of 36 studies reported that psychological stress, particularly chronic stress, negatively influences specific stages of IVF, with the egg retrieval stage being most vulnerable and fertilization also affected to a lesser extent. Follicular cortisol was identified as an important biological marker impacting multiple IVF stages, supporting a mechanistic link between HPA‐axis activation and reproductive outcomes [33]. Meditation‐based interventions have been reported to enhance HPA axis regulation, which may attenuate the detrimental effects of mood disturbances on reproductive outcomes. Although some studies suggest that meditation may increase pregnancy rates, the findings are inconsistent and appear to depend on study design and the type and duration of meditation practice. For instance, one study reported that very brief meditation protocols, either a total of 80 min over 2 weeks or daily 15‐min sessions for 2 weeks, did not result in a significant improvement in pregnancy outcomes during ART cycles [29]. This variability in the different study results highlights the need for further research examining the effect of specific mindfulness‐based interventions on mental health and pregnancy outcomes of infertile women.

### 4.1. Limitations

This study has several limitations that should be considered when interpreting the findings. First, the study population consisted solely of Iranian women, which may limit the generalizability of the results to infertile women from other cultural or ethnic backgrounds. Second, the follow‐up period was limited to 3 months, so the long‐term sustainability of the observed effects on psychological distress and pregnancy outcomes remains unknown. Third, the study was not blinded, which could have introduced performance or expectation bias among participants and clinicians. Fourth, the sample size was relatively modest, reducing statistical power and potentially limiting the detection of smaller, but clinically meaningful effects. Additionally, adherence to home‐based MBSR practice was self‐reported, which may have introduced reporting bias. Finally, other unmeasured factors, such as variations in IVF protocols or underlying infertility etiology, could have influenced the outcomes.

## 5. Conclusions

This study provides some evidence for the efficacy of online MBSR intervention as a complementary therapy for controlling emotional distress and improving pregnancy rate in infertile women undergoing IVF. Online MBSR classes can be used as a promising communicative tool for enabling women undergoing IVF to reduce their infertility‐related mental distress. Integrating structured stress‐reduction interventions such as online MBSR programs into routine IVF care provides clinicians with a practical, nonpharmacological tool to support patients’ mental health. Such interventions can empower women to actively manage stress, enhance coping strategies, and maintain engagement with complex fertility procedures. Moreover, offering online formats increases accessibility for patients who may face geographic, time, or mobility constraints, making psychological support more inclusive and scalable in fertility clinics. By addressing the psychological dimension of infertility care, clinicians can adopt a more holistic approach that aligns with patient‐centered practice and potentially improves overall treatment experiences.

## Author Contributions

A.K.V., R.S., S.A., and S.M. contributed to the study concept, design, data collection, literature search, and manuscript revision. M.M. and S.H.N. contributed to the design, data analysis and interpretation, literature search, interpretation, and revision of the manuscript. F.N. contributed to the study design, data collection, and manuscript revision.

## Funding

The authors declared that this study has received financial support from a grant from Shiraz University of Medical Sciences (Grant No. 20149).

## Disclosure

All the authors mentioned above approved the final manuscript.

## Ethics Statement

This study was reviewed and approved by the Ethics Committee of Shiraz University of Medical Sciences (Approval No. IR.SUMS.MED.REC.1400.310). All participants provided written informed consent before participation, and the study was conducted in accordance with the Declaration of Helsinki and relevant national guidelines on human research ethics.

## Consent

Please see the Ethics Statement.

## Conflicts of Interest

The authors declare no conflicts of interest.

## Data Availability

The datasets generated and analyzed during the current study can be obtained from the corresponding author upon reasonable request.
